# New technologies in anesthesia and intensive care: take your ticket for the future

**DOI:** 10.1186/s44158-023-00098-3

**Published:** 2023-05-31

**Authors:** Elena Bignami, Roberto Lanza, Giacomo Cussigh, Valentina Bellini

**Affiliations:** Anesthesiology, Critical Care and Pain Medicine Division, Department of Medicine and Surgery, University of Parma, Parma, Italy

**Keywords:** New technologies, Artificial intelligence, Metaverse, Anesthesia, Big data, Machine learning, Telemedicine, Internet of medical things, Block chain

## Abstract

The modern world runs all around hi-tech, which surrounds us in our everyday life. The medical field is no less; the introduction of the novel disruptive technologies are transforming every healthcare system. Anesthesia, intensive care, and pain medicine are fields in which the application of new technologies is proving to have great potential. However, it is crucial that this digital medical transformation always takes place under the coordination of natural (human) intelligence.

As William Gibson wrote: “The future is already here—It’s just not very evenly distributed”, What is happening in this historical phase of ours is somewhat paradoxical; while everyday tools, such as phones or smartwatches, are equipped with very advanced technologies, that are constantly update, some of them to the point of being invisible to our eyes. Instead in our professional life we use tools that, when compared to the previous ones, can be considered obsolete sometimes. Fortunately, this trend is changing and as healthcare professionals we must demand a rapid evolution, albeit within the limits of patient safety and ethics respect. The figure shows us that today everything is interconnected, both for the various branches that orbit around the anesthesia’s world both for the technologies and tools that are used. There are no more watertight compartments, it is all a cloud from which they draw, share and renew ideas, assets, and technologies. Big data are an example, for which ICU was the ideal environment due to the large amount of information collected and processed; nowadays is easy do likewise in other specialties.

It is time to buy the ticket and get on the train of the future (Fig. 1): jump aboard the red AI’s train and see that there are no limits to its application into the medical field, from the improvement of resource management, going to an early identification and prediction of patients at risk of sepsis, to an Hypotension Prediction Index based on algorithm derived from machine learning that uses the arterial waveform to predict intraoperative hypotension [1]. Or get the yellow wearable devices’ train, which shows tools that gaining biosignlas such as heart rate, respiratory rate, skin temperature, and much more [2]. This not only will help the clinician to monitor and provider rapid healthcare actions but is going to have a promising way to the pain assistance and management too. Even there are different itinerary, every specialty could benefit from all the features within the map [3]. The tools (new technologies, NT) that enable us to embark on this journey (big data, telemedicine, artificial intelligence and machine learning, block-chain technologies, Internet of medical things, and the latest addition: metaverse) seem to now have the characteristics making them adaptable to our daily clinical practice. Some of these paths, fortunately, have already started to be traced, representing the oldest lines, as in ICU and perioperative medicine [4, 5]. Others, such as emergency, pain medicine, and organizational settings, are opening up. One rule must always be respected: the trains will have to move throughout the gray metropolitan area, represented by a human brain.

**Fig. 1:**
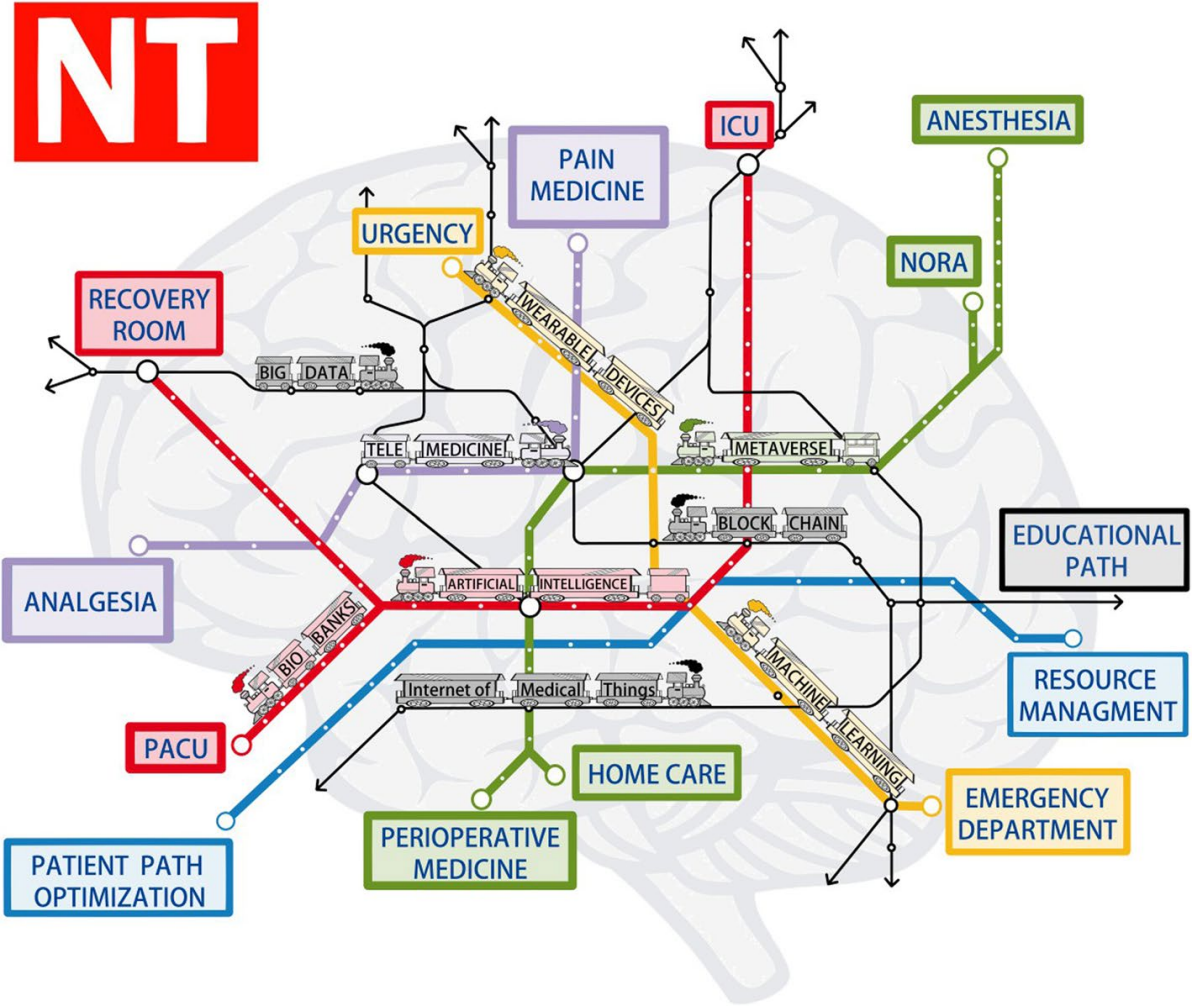
The figure shows the map of new technologies(NT) in which every line is interconnected, thereby every tool (colored trains) is available for each line. The human brain in the background tells that is essential the human factor in order to use the NT to create a hybrid intelligence

It is essential that NT always interact with human intelligence in a constant interconnection, creating a new super hybrid clinical model, bearing in mind that NT are not meant to take the place of clinicians, as in the famous dystopian movies, but are handy tools more or less complex to support, simplify, and enhance their job.

The ticket is cheap and allows you unlimited access all over the map, buy it!

## Data Availability

Not applicable.
